# On sorption hysteresis in wood: Separating hysteresis in cell wall water and capillary water in the full moisture range

**DOI:** 10.1371/journal.pone.0225111

**Published:** 2019-11-15

**Authors:** Maria Fredriksson, Emil Engelund Thybring

**Affiliations:** 1 Division of Building Materials, Department of Building and Environmental Technology, Lund University, Lund, Sweden; 2 Biomass Science and Technology, Forest Nature and Biomass, Department of Geosciences and Natural Resource Management, University of Copenhagen, Frederiksberg, Denmark; Bartin University, TURKEY

## Abstract

Moisture influences most physical wood properties and plays an important role in degradation processes. Like most other porous materials, wood exhibits sorption hysteresis. That is, the moisture content is higher if equilibrium is reached by desorption than if it is reached by absorption under the same ambient climate conditions. The mechanism of moisture uptake by wood are different in the hygroscopic and over-hygroscopic moisture ranges and due to methodical issues, most studies of sorption hysteresis have been performed in the hygroscopic range. In the present study, total sorption hysteresis was separated into hysteresis in cell wall water and capillary water respectively in the whole moisture range by a novel combination of experimental techniques. Wood specimens were conditioned to several high moisture contents using a new system based on the pressure plate technique, and the distinction between cell wall water and capillary water was done with differential scanning calorimetry. The results showed that sorption hysteresis in wood cell walls exists in the whole moisture range. The cell walls were not saturated with water until the whole wood specimen was saturated which contradicts the long-held dogma that cell walls are saturated before significant amounts of capillary water are present in wood.

## Introduction

Moisture influences a range of important properties of wood such as mechanical behaviour and dimensional stability and additionally plays an important role in degradation processes. Therefore, the relation between wood and moisture has been widely studied within several fields of research, e.g. pulp and paper [1, 2], bio-refining [3, 4], wood science [5, 6], and civil engineering [7, 8].

Within the wood material, moisture can be present both in cell walls and as capillary water in macro-voids such as cell lumina and pit chambers. Water molecules inside cell walls interact with hydroxyl groups of the constituting polymers (cellulose, hemicellulose and lignin) by hydrogen bonding while moisture in macro-voids is predominantly found as capillary condensed water. The dimensions of these macro-voids are on the micrometre scale and therefore, high relative humidity levels are required for capillary condensation to yield significant amounts of water here.

Like most other porous materials, wood exhibits sorption hysteresis. That is, the moisture content does not only depend on the ambient climate (relative humidity and temperature), but also on the moisture history; the moisture content is higher if equilibrium is reached by desorption than if it is reached by absorption under the same ambient climate conditions [5, 9]. Since the mechanisms of moisture uptake are different inside and outside of cell walls, the mechanisms behind sorption hysteresis suggested in literature are also different. For water in macro-voids, the main suggested mechanism is the ink-bottle or pore blocking effect [9, 10] where pits act as bottlenecks to the emptying of cell lumina during desorption. For sorption hysteresis in cell walls, several possible mechanisms have been put forward relating to the mechanical response of cell walls and hydrogen bonding configurations as the dimensions change due to a change in moisture content [11–13].

The presence of capillary water in macro-voids is often taken as an indication that cell walls are saturated with water [14–16]. This is deduced from the fact that at high moisture levels, the mechanical properties and bulk dimensions of wood do not depend significantly on the moisture content, whereas these properties strongly depend on moisture content at lower moisture levels [14, 17–19]. The moisture content at the infliction point between these two ranges is commonly referred to as the fibre saturation point [14]. Over time, several additional definitions of the fibre saturation point have been presented, see reviews in [11, 20, 21]. Most of these rely on the assumption that cell walls are saturated before the whole piece of wood is saturated with water. In this picture of water sorption in wood, sorption hysteresis at moisture contents close to saturation would be ascribed solely to hysteresis in amount of capillary water. There are, however, differences in reported values of cell wall moisture content determined for water saturated specimens by solute exclusion, Low Field Nuclear Magnetic Resonance (LFNMR) or Differential Scanning Calorimetry (DSC), and the fibre saturation point derived from mechanical properties or bulk dimensions; the latter is generally lower [11]. This indicates that cell walls are not saturated when the change in mechanical properties and bulk dimensions occurs, and it has therefore been suggested that fibre saturation should be seen as a gradual transition rather than a fixed moisture content [11]. Also, dimensional changes in wood have been observed when capillary water was still present in the wood [17–19]. It is therefore possible that sorption hysteresis at humidity levels close to saturation, is partly due to cell wall sorption hysteresis. Recently, the absolute sorption hysteresis, i.e. the absolute difference between equilibrium moisture content in desorption and absorption, was shown to increase linearly with relative humidity in the hygroscopic moisture range up to 95% relative humidity [5]. Less is, however, known about sorption hysteresis in cell walls in the over-hygroscopic moisture range, i.e. the moisture range above 95–98% relative humidity.

Sorption hysteresis is commonly determined by conditioning specimens in both absorption and desorption to several relative humidity levels. Sorption balances or conditioning above saturated salt solutions are widely used methods for this purpose, e.g. [5, 22, 23], but they cannot be used above relative humidity levels of 95–97%. Above these levels, i.e. in the over-hygroscopic moisture range, other methods such as the pressure plate technique is needed, see e.g. [19, 24, 25]. Since this method was designed for desorption measurements, performing absorption measurements requires adjustments of the experimental set-up [24]. Therefore, few measurements of absorption isotherms and studies of sorption hysteresis are available in the over-hygroscopic range, but previous work on wood includes studies on western hemlock (*Tsuga heterophylla* (Raf.) Sarg.) [9], Norway spruce (*Picea abies* (L.) Karst.) [24], spruce [26] and aspen (*Populus tremuloides* Michx.) [27]. However, in all these studies, hysteresis in total moisture content was determined, i.e. the location of water within the wood structure was not taken into account.

Differential Scanning Calorimetry (DSC) enables evaluation of the amount of freezable and non-freezable water by exposing a specimen to sub-zero temperatures, increasing the temperature and determine the enthalpy of melting [28]. Based on the assumption that cell wall water is non-freezable in the temperature range employed in the DSC measurements, water in cell walls and water in macro-voids can be separated. In previous studies, this method has been used to determine cell wall moisture content of water saturated specimens [28–31]. Water tightly associated with polymeric materials such as extracted cellulose and lignin can, however, freeze when the temperature falls well below the normal freezing point of liquid water [32, 33]. This freezable water is presumably found in clusters around strongly polar groups [34, 35]. Freezable water is not found in solid wood cell walls, but can be found in powdered wood samples where water in small voids between particles can freeze at a depressed freezing point compared with liquid water [29].

In the present study, total sorption hysteresis was for the first time separated into hysteresis in cell wall water and capillary water respectively in the full moisture range. A novel pressure plate system, enabling conditioning in both absorption and desorption at several pressure levels simultaneously, was used in the over-hygroscopic moisture range. In addition, to cover the full moisture range, specimens were also conditioned above saturated salt solutions. After conditioning to equilibrium, DSC was used to separate cell wall water and capillary water.

## Pressure plate conditioning system

### Background

In the pressure plate technique, specimens are placed on a porous ceramic plate in a pressure vessel, and gas pressure is applied. The applied pressure will induce a curvature of the water menisci in the air-water interface in the pores in the ceramic plate, which will define the humidity in the pressure vessel to which the specimens will eventually equilibrate. The relation between the applied pressure and relative humidity, *φ* (Pa Pa^-1^), is given by:

$$\text{ln}\left(\varphi \right)=-\frac{\Delta PM_{w}}{RT\rho_{w}}$$ (1)

where Δ*P* (Pa) is the pressure relative to the atmosphere pressure, *M*_w_ (0.018 kg mol^-1^) is the molar weight of water, *R* (8.314 J mol^-1^ K^-1^) is the gas constant, *T* (K) is temperature, and *ρ*_w_ (kg m^-3^) is the density of water. The moisture state can also be given as water potential, *ψ* (J kg^-1^), which is related to relative humidity by:

$$\psi =\frac{RT}{M_{w}}\text{ln}(\varphi )$$ (2)

For more details on the theoretical background, see [36].

Often, large pressure vessels are used for conditioning many specimens at the same time. However, since the time to equilibrium commonly is in the range of two months, it is time-consuming to condition specimens to several moisture levels. In the present study, a pressure plate system with several smaller pressure plate cells was developed to enable conditioning in 20 cells at ten different pressure levels at the same time. The cells were designed to enable measurements both in absorption and desorption.

### Experimental set-up

The pressure supply system consisted of 10 pressure regulators (0–6.8 bar, KPR1FJA412A20000, Swagelok Company, Solon, OH, USA) and 10 pressure sensors (S-20 0–6 bar, WIKA, Lawrenceville, GA, USA) and pressure was supplied by a nitrogen generator. The maximum pressure of the pressure plate cells were 5 bar and the range of measurement was thus 0–5 bar, corresponding to the relative humidity range 99.6–100%. Two pressure plate cells were connected to each pressure regulator to enable measurements of both absorption and desorption at the exact same pressure. Both cells could however be run in either desorption or absorption. Each cell was connected by a quick connect (SS-QC4-B-6M0 and SS-QC4-D-2PM, Swagelok Company, Solon, OH, USA) which enabled each cell to be disconnected from the pressure supply system while keeping the cell under pressure.

**Fig 1:**
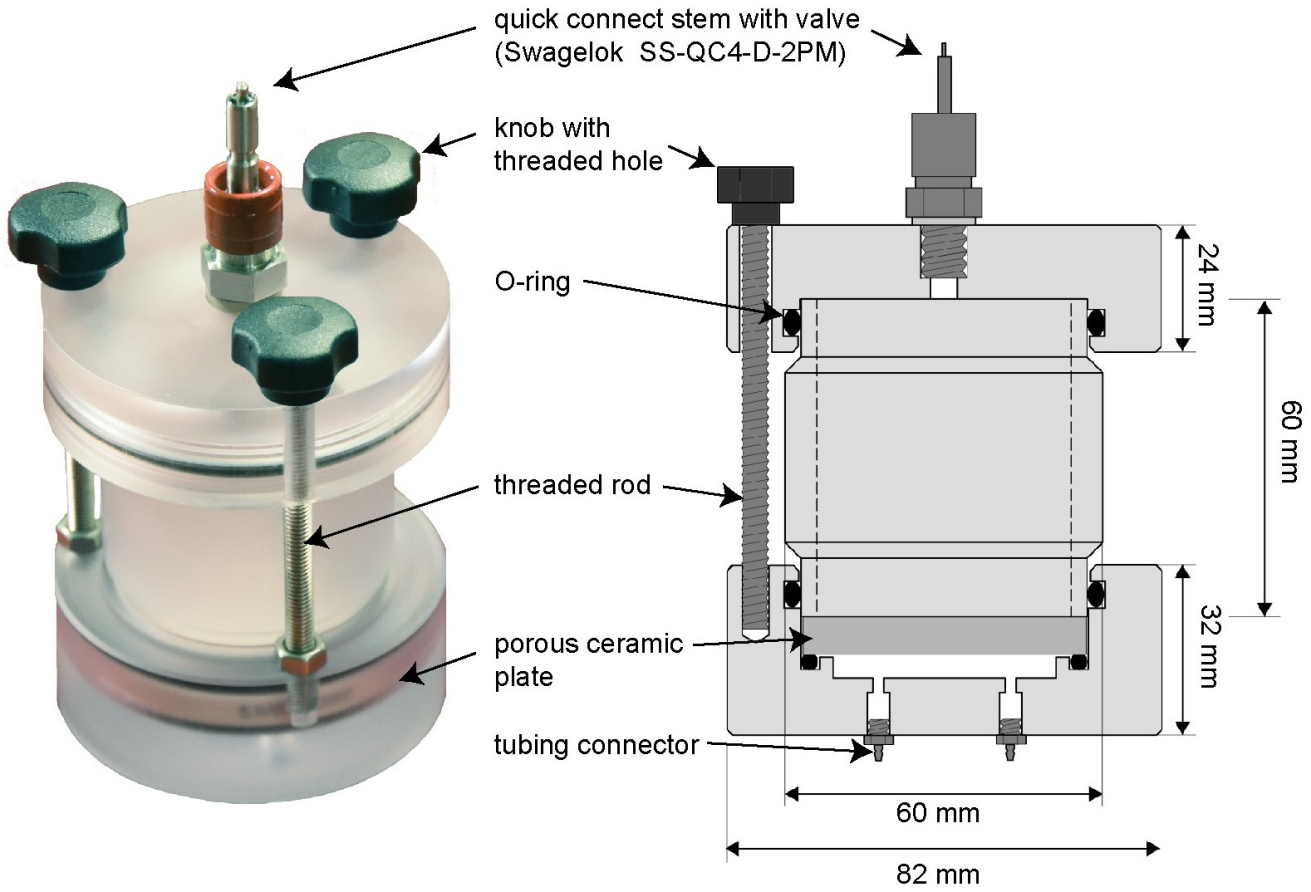
Design of pressure plate cell. Photo of a pressure plate cell (left) and a section through the middle of the cell (right).

The design and dimensions of one pressure plate cell is shown in Fig 1. The cell consisted of three parts made in polycarbonate which could be separated from each other: a bottom part, a middle cylinder and an upper lid. The bottom part included a porous ceramic plate (2.0” diameter, 5 Bar standard, Soilmoisture Equipment Corp., Santa Barbara, CA, USA), which was mounted between O-ring seals. To allow water circulation, there was a space of 4 mm below the ceramic plate with one inlet and one outlet. The cell was held together by three threaded rods, which were attached to the bottom part of the cell. The upper lid was fastened by knobs with threaded holes.

**Fig 2:**
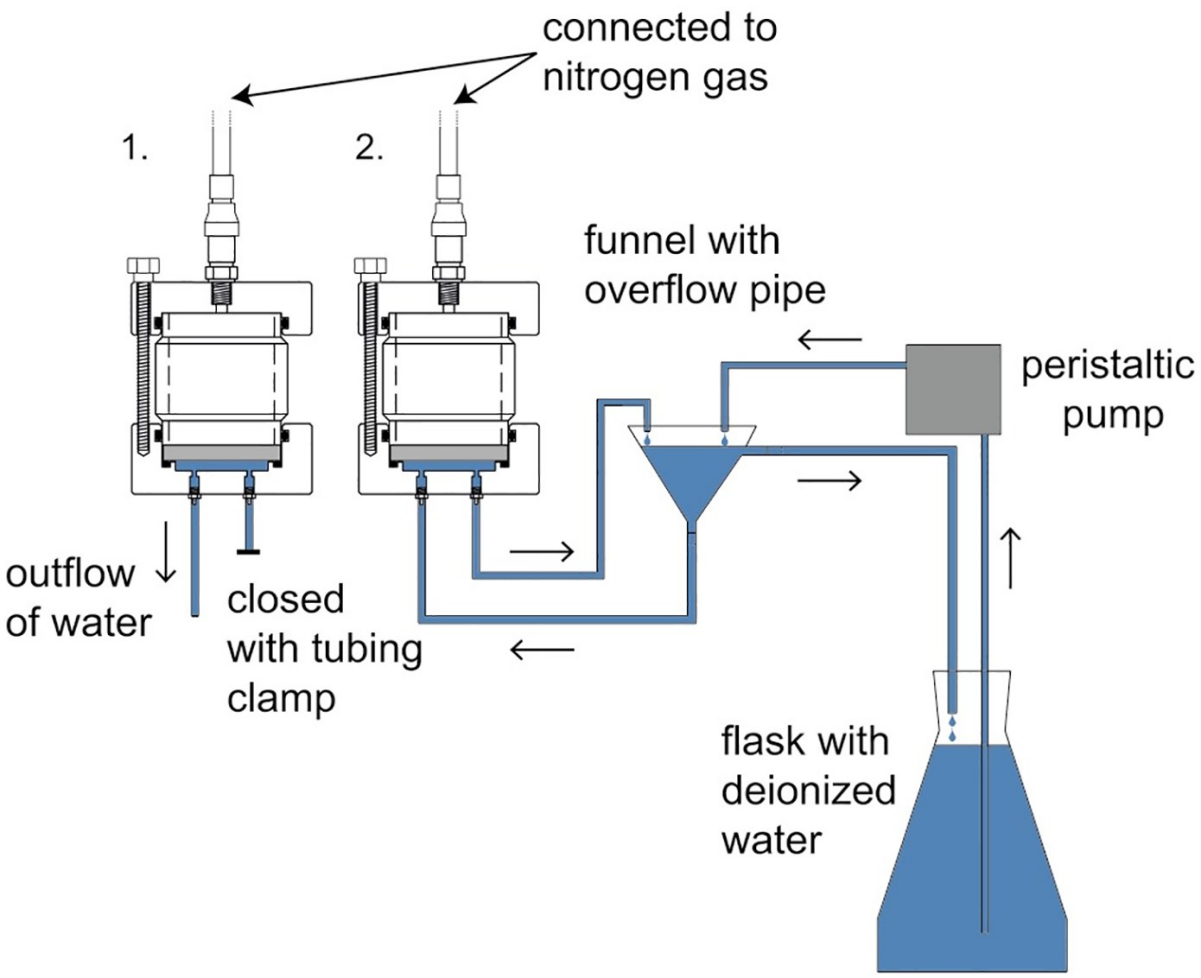
Experimental set-up pressure plate. A schematic illustration of the experimental set-up and the water supply system for the absorption pressure plate cells.

Absorption measurements require that water is supplied during the experiment. In a previous study reported by [24], water was supplied directly to the space below the ceramic plate by peristaltic pumps. However, using this method, care must be taken so that the peristaltic pumps do not build up pressure below the porous ceramic plate. In the present study, water was instead supplied by the principle of communicating vessels. For absorption measurements, the inlet on the bottom part of the cell was connected to the stem of a glass funnel (Fig 2). This funnel was provided with deionized water from a flask by a miniature peristaltic pump (WPM1, WELCO Co. Ltd., Tokyo, Japan). On the side of the upper part of the funnel, a short glass pipe had been added and the water level in the funnel could therefore not exceed this level. Instead, when the funnel was full, water was lead back to the flask (Fig 2). The funnel was positioned so that the level of the glass pipe on the side was slightly above the level of the underside of the porous ceramic plate. The outlet of the pressure plate cell was connected to tubing which lead water back to the funnel (Fig 2). In this way, the water supply was a closed system, but without the risk of building pressure below the ceramic plate. The top of the flask with deionized water was partly covered with parafilm to decrease evaporation.

## Materials and methods

### Material

Douglas fir (*Pseudotsuga menziesii* (Mirb.) Franco) was sawn into samples of approximate dimensions 80 x 40 x 2 mm^3^ where the shortest dimension was in the longitudinal direction. To remove extractives that might influence the results, the samples were extracted in a Soxhlet apparatus, first in ethanol and toluene (ratio 1:2) for 24 h and secondly in acetone and MilliQ water (ratio 9:1). The dry density after extraction was about 540 kg/m^3^ and the growth ring width about 2 mm with about 35% latewood. Circular discs with an approximate diameter of 4 mm and a thickness of 2 mm (longitudinal direction) were cut using a razor blade. In addition, all measurements were performed on unextracted material, see S1 Appendix.

### Over-hygroscopic moisture range

Sorption hysteresis and sorption isotherms in the over-hygroscopic moisture range were determined by conditioning specimens by the pressure plate system described above. After conditioning, DSC measurements were performed on the specimens in order to separate water in cell walls and capillary water. The experimental procedure is described in detail in the following sections.

#### Conditioning

A total of 60 specimens were dried in a vacuum oven at 60°C for 24 h. Thereafter, 25 specimens were stored above molecular sieves (0.4 nm, Merck, Darmstadt, Germany), and 35 other specimens were vacuum saturated with deionized water. This was done by keeping the specimens in a desiccator at 2 mbar for 2.5 h before water was let in (pressure hereby increased to 40 bar). After an additional 30 min, atmospheric pressure was reinstated, causing the specimens to immediately sink. The specimens where kept in water for 6 days. The water-saturated mass was determined for each specimen after gently wiping off excess surface water with a moist cellulose based cloth (Wettex, Vileda, Freudenberg Home & Cleaning Solutions AB, Malmö, Sweden). The bottom part of five pairs of absorption and desorption pressure plate cells were also vacuum saturated with deionized water.

For desorption measurements, the water inlet tubing in each cell was plugged by a tubing clamp and the outlet tubing was led to a small measuring cylinder. The desorption pressure plate cell was assembled by attaching the polycarbonate cylinder to the bottom part of the pressure plate cell, five water saturated specimens were placed on the water-saturated ceramic plate, and the top lid was fastened. For absorption measurements, the water inlet and outlet were connected to the water supply system as shown in Fig 2 and the tubing was filled with deionised water. To assembly the absorption pressure plate cell, the cylinder was attached to the bottom part of the cell and a grid was placed on the ceramic plate. The grid was made of aluminium and was attached to an aluminium cylinder with a height of 20 mm and a diameter of 45 mm. Five dried specimens were placed in each cell on top of the grid to avoid contact with the water-saturated ceramic plate. Subsequently, the lid was attached to the cell and each cell was connected to the pressure supply system. Finally, the intended pressures were applied and the peristaltic pump was started. In addition, five water-saturated specimens were conditioned above a saturated solution of potassium nitrate (KNO_3_) which generates a relative humidity of 94.6% [37]. All conditioning were performed for 2 months at 20.4 ±0.3 °C (spikes of short duration occurred at some occasions). For this period of time, the remaining five water-saturated specimens were kept in water in a refrigerator. The long conditioning time was chosen to be sure that equilibrium was reached for the pressure plate conditioned specimens. This time exceeded the 5 weeks needed for larger and thicker specimens (5 mm) to reach equilibrium in normal pressure plate cells (see S1 Appendix). An overview of the moisture levels, conditioning methods and number of specimens included is given in Table 1.

**Table 1:** Overview of moisture levels, conditioning methods and number of specimens used for determination of sorption hysteresis and sorption isotherms.

Pressure (bar)	Salt solution	RH (%)	Water potential (J kg^-1^)	Cond. method	No. of specimens	
					*absorption*	*desorption*
***Over-hygroscopic range***						
		100%	0.00	VS	-	5
0.33	-	99.98%	-27.05	PP	5	5
0.93	-	99.93%	-94.69	PP	5	5
1.22	-	99.91%	-121.76	PP	5	5
3.01	-	99.78%	-297.82	PP	5	5
4.81	-	99.65%	-474.11	PP	5	5
-	KNO_3_	94.6%	-7 506.62	SS	-	5
***Hygroscopic range***						
-	KNO_3_	94.6%	-630 233.33	SS	5	5
-	KCl	85.1%	-644 544.08	SS	5	5
-	NaCl	75.5%	-660 729.56	SS	5	5
-	NH_4_NO_3_	64.4%	-682 232.65	SS	5	5
-	Mg(NO_3_)_2_	54.4%	-705 051.53	SS	5	5
-	MgCl_2_	33.1%	-772 234.68	SS	5	5

The conditioning was performed by vacuum water-saturation (VS), pressure plate (PP) or saturated salt solutions (SS).

#### DSC measurements

After conditioning for two months, the conditioned and water-saturated specimens were transferred to DSC pans by the following procedure. A moisture generator (2500 Humidity Generator, Thunder Scientific Corporation, Albuquerque, New Mexico, USA) was connected to a glovebox to generate a high relative humidity (> 95%) inside the glovebox. Masses of all DSC pans (Tzero pan and Tzero hermetic lid, TA Instruments, New Castle, DE, USA) were determined with a resolution of 0.1 mg. The pressure plate cells were detached from the pressure supply system and moved into the glovebox with pressure remaining inside the cells. For one cell after another, the pressure was released and one specimen was placed in each DSC pan that was hermitically sealed inside the glovebox using a Tzero press (TA Instruments, New Castle, DE, USA). Finally, the mass of each DSC pan including specimen was determined. The same procedure was used for specimens conditioned above potassium nitrate and water-saturated specimens. The water-saturated specimens were, however, wiped with a moist cellulose based cloth to remove excess surface water before they were placed in the DSC pans.

The DSC measurements were undertaken using a DSC Q2000 (TA Instruments, Eschborn, Germany) with an autosampler. Measurements were performed in three batches and a total of six reference pans with water were used. Each measurement was performed as follows [31]: The temperature was quenched to -20 °C and held constant for 5 min before ramping with 2 °C min^-1^ to 20 °C. Finally, the temperature was raised to 40 °C before the autosampler changed DSC pans. When all runs were completed, the lids of the DSC-pans were pierced multiple times with a syringe needle before the pans with specimens inside were dried in a vacuum oven at 60 °C for 24 hours. Finally, the pans were cooled in containers with molecular sieves before their dry masses were determined. The total moisture content, *ω*, of each specimen was evaluated by:

$$\omega =\frac{m_{eq}-m_{dry}}{m_{dry}}$$ (3)

where *m*_eq_ (g) is the mass of the specimen conditioned to a certain moisture level, and *m*_dry_ (g) is the dry mass of each specimen. The moisture content related to capillary water *ω*_cap_ (g g^-1^) was determined as the amount of freezable water. In order to calculate this quantity, the total heat of fusion *Q* (J g^-1^) was determined by integration of the melting peak in a temperature interval visually picked from the heating curves in the software TA Universal Analysis 2000 (version 4.5A, TA Instruments, Eschborn, Germany). Based on the total heat of fusion, the dry wood mass (*m*_dry_) and the moist wood mass (*m*_eq_), the cell wall moisture content, *ω*_cw_, was found as:

$$\omega_{cw}=\frac{m_{eq}-m_{dry}-(\frac{Q∙m_{eq}}{H_{f}})}{m_{dry}}$$ (4)

where *H*_f_ (J g^-1^) is the enthalpy of fusion of water of 333.7 J g^-1^. Calibration of the DSC Q2000 for enthalpy of fusion was done with deionised water (melting point 0 °C, enthalpy of fusion 333.7 J g^-1^). The capillary water content was determined as the difference between *ω* and *ω*_cw_.

Finally, the absolute sorption hysteresis in cell wall water and capillary water, respectively, was evaluated as the difference between the moisture content in desorption and absorption at each relative humidity level. The standard deviation of the sorption hysteresis was evaluated as the standard deviation of the difference between sample means.

### Hygroscopic moisture range

A total of 60 specimens were dried in a vacuum oven at 60 °C for 24 hours. Thereafter, 30 of these specimens were water-saturated using the vacuum procedure described above. Six saturated salt solutions (see Table 1) were prepared and placed in small climate boxes (170 mm x 250 mm x 100 mm) with a relative humidity sensor and a small fan for air circulation. 30 dried specimens and 30 water-saturated specimens were placed in the six boxes, i.e. 5 dried and 5 water-saturated specimens were placed in each box. The specimens were kept in the boxes for 2 months. Subsequently, the mass of each specimen was determined by the following procedure. One of the climate boxes was placed in a glove box to which a moisture generator (2500 Humidity Generator, Thunder Scientific Corporation, Albuquerque, New Mexico, USA) was connected. The level of relative humidity inside the glovebox was set to that in the climate box. When the relative humidity was stable, the climate box was opened and the mass of each specimen was determined on a balance inside the glovebox. This procedure was repeated for all relative humidity levels and climate boxes. All specimens were finally dried in a vacuum oven at 60 °C for 24 hours, and the dry masses were determined with a resolution of 0.1 mg. The moisture content was evaluated by Eq 3 and absolute sorption hysteresis was evaluated as described above.

## Results

**Fig 3:**
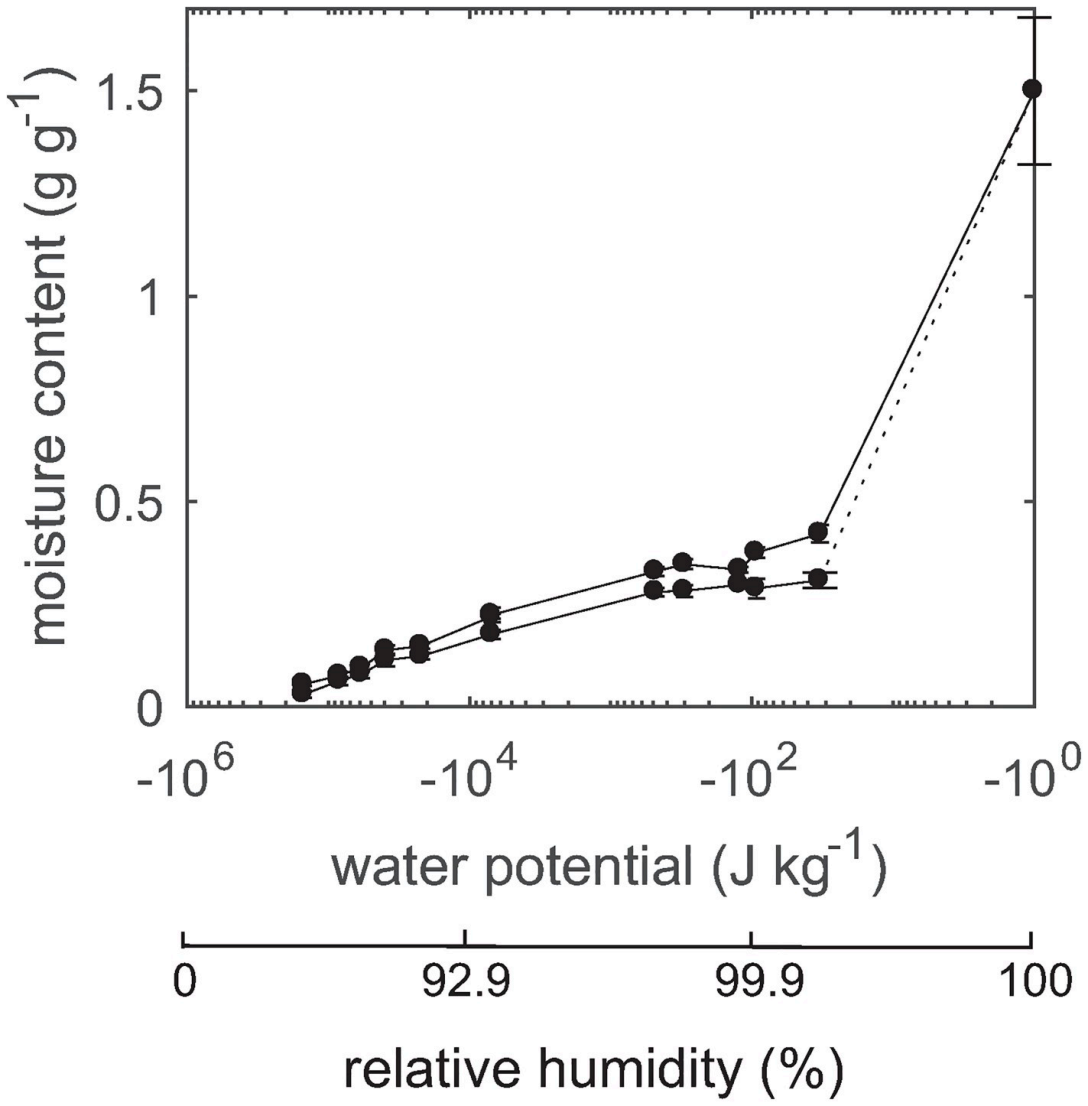
Total sorption isotherms. Total absorption (lower) and desorption (upper) isotherms.

**Fig 4:**
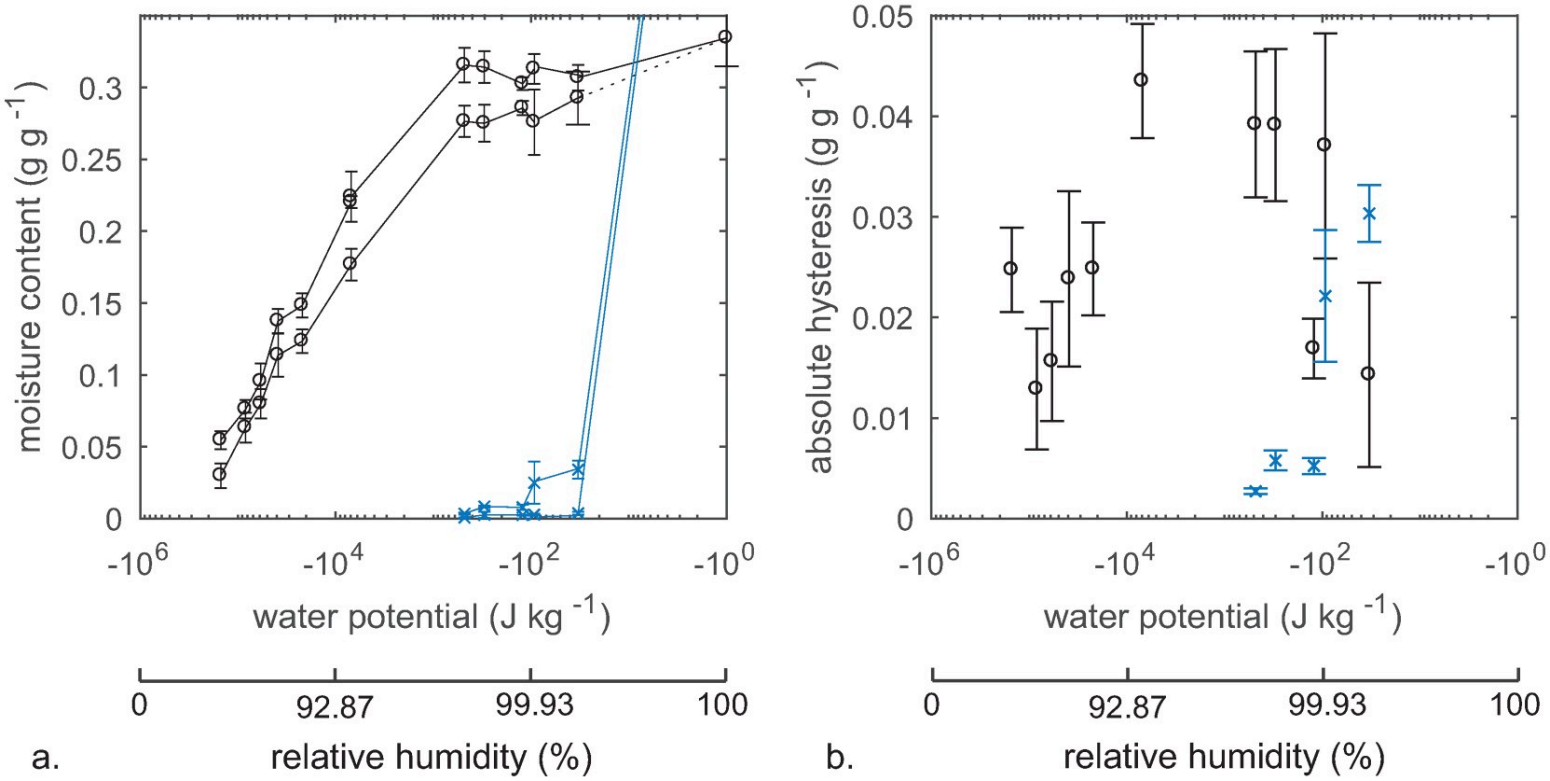
Separated sorption isotherms and sorption hysteresis *a*. Cell wall sorption isotherms (o) and capillary water isotherms (x). The y-axis is cut, but the capillary moisture content at water saturation was 0.792 g g^-1^ with standard deviation 0.215 g g^-1^. Note that moisture contents at water-saturation are placed at -10° J kg^-1^. *b*. Absolute sorption hysteresis evaluated from the data in *a*.

Fig 3 shows full sorption isotherms obtained by pressure plate conditioning and conditioning above saturated salt solutions. The DSC measurements enabled separation of capillary water and cell wall water and the cell wall absorption and desorption isotherms were evaluated in the whole moisture range (Fig 4a). As expected, capillary water was not detected for specimens conditioned at 95% RH and therefore DSC measurements were not performed for specimens conditioned to relative humidity levels below 95%. All cell wall moisture contents obtained by conditioning in absorption was lower than the moisture content at saturation (Fig 4a). This pattern was also seen for the cell wall moisture contents obtained by conditioning in desorption although the difference was not as distinct as for absorption.

Sorption hysteresis in cell wall water was seen also in the over-hygroscopic range; the cell wall desorption and absorption isotherms did not merge until in the water-saturated state (Fig 4b). Fig 4b shows that cell wall sorption hysteresis increased up to 95% RH, but indicates a decrease in the over-hygroscopic moisture range. In contrast, hysteresis in capillary water, increased in the over-hygroscopic range (Fig 4b).

Additional results for unextracted material is found in S1 Appendix.

## Discussion

### Sorption hysteresis

The absolute sorption hysteresis increases in the hygroscopic moisture range [5] and the results from the present study show that sorption hysteresis persists in the over-hygroscopic range; the absorption and desorption isotherms only merge in fully water-saturated state (Fig 3) as also seen in previous studies [9, 24, 26, 27]. Total sorption hysteresis was smaller in the over-hygroscopic range than in previous studies [9, 24, 27] which is due to the thin specimens used in the present study. Since the specimen thickness was smaller than the length of a tracheid cell, which is normally 3–4 mm in Douglas fir [38, 39], the pits did not act as ink-bottle necks and the water present in cell lumina was emptied at a higher relative humidity than for samples with a thickness exceeding 3–4 mm. Consequently, the upper part of the total desorption isotherm in Fig 3 is slightly lower than for thicker specimens The amount of capillary water and hysteresis in capillary water should thus be interpreted with care and is not representative for amount of capillary water in thicker specimens. The specimen thickness was, however, limited by the height of the DSC pans used, and the main focus of the present study was to study cell wall sorption hysteresis. In S1 Appendix, total desorption isotherms obtained for specimens with thicknesses of 5 and 2 mm are shown together with the data from Fig 3.

Looking only at water in cell walls, the results of the present study indicate that sorption hysteresis increases in the hygroscopic range but decreases in the over-hygroscopic range. The former is in line with the observations of [5], while this study provides the first-ever data on cell wall sorption hysteresis in the over-hygroscopic moisture range.

The presence of increasing amounts of liquid water in the macro-void wood structure appears to decrease the cell wall sorption hysteresis as seen in Fig 4b. Thus, it can be speculated whether liquid water held in macro-voids can re-open the cell wall structure better than water vapour. This would be in line with results by [40] who used deuterium oxide to investigate the amount of hydroxyl groups interacting with water within wood cell walls. In that study, liquid water was able to re-open and interact with more hydroxyls than after conditioning to 95% relative humidity. At that level, Fig 4b of the present study shows the cell wall sorption hysteresis to be at its maximum. Beyond this relative humidity level, the difference in cell wall moisture between wood conditioned in absorption and desorption is gradually decreasing, perhaps due to re-opening of the cell wall structure.

### The fibre saturation point concept

Fig 4 shows that the presence of capillary water in macro-voids does not indicate that cell walls are water-saturated; the cell wall moisture content was lower at 99.98% relative humidity than at water-saturation in both desorption and absorption, although the difference was more pronounced in absorption (Fig 4a). This contradicts the original concept of the fibre saturation point where cell walls are suggested to be saturated with water before significant amounts of capillary water are present in wood [14–16]. The fibre saturation point concept have previously been questioned based on observations of liquid water within the wood structure after a decrease in the cell wall moisture content [41–43]. These observations were made either during drying or by conditioning to well-defined moisture states by desorption. However, drying inevitably causes moisture gradients within specimens, and therefore, experimental data obtained during drying to a non-equilibrium state are not suitable for evaluation of location of water at a certain moisture content. For the studies that observed liquid water after specimens were conditioned to well-defined moisture states by desorption [42, 43], this was attributed to pore bottlenecks in the wood micro-structure, i.e. water was held in cell types with small pit openings. The present study documents, however, for the first time that cell walls are not water-saturated at any point in the over-hygroscopic range below full water-saturation of the wood. Even as high as 99.98% relative humidity, the cell wall moisture content was lower than at full water-saturation by 2% in desorption and 4% in absorption. This illustrates that it is not possible to condition wood by water vapour to a moisture state, where cell walls are saturated, whether there is liquid water in the macro-void structure or not. Thus, the results also explain why the fibre saturation point derived from mechanical properties or changes in bulk dimensions is lower than the cell wall moisture content determined on fully water-saturated specimens.

### Assignment of freezing water to capillary water

In this study, the separation of the moisture content into cell wall water and capillary water held in the macro-void wood structure was done based on the DSC measurements. Thus, it is assumed that in the temperature range -20 °C to 20 °C, the cell wall water does not exhibit a phase change while the capillary water does. This assumption is based on several arguments. Firstly, previous studies have shown that freezing cell wall water is only found in cellulosic materials in association with strongly polar groups [34, 35]. Even though some studies have reported freezable water in association with samples of extracted cellulose and lignin [32, 33], any observed depressed phase change in powdered wood has been shown to be due to water held in small voids between particles [29]. Thus, for solid samples of wood no freezing cell wall water has been found [29]. Secondly, cell wall water is held in very small pores within the material of the order of 2 nm [44–46], and would therefore be expected to have a markedly depressed phase change around -30 °C [47, 48]. However, in our measurements, the melting peak always occurred close to the melting point of pure water, indicating that the melting energy does not derive from any phase change in cell wall water.

Nonetheless, it could be of interest to investigate how the results would be different if our assignment of non-freezing and freezing water was not fully correct. If there were freezing water within the cell walls, contrary to the results of [29, 34, 35], the DSC method would underestimate the cell wall moisture content. We would expect that the underestimation would be higher with higher amount of cell wall water, since higher cell wall moisture content would produce either larger or more water-filled pores. As a result, the difference in cell wall moisture content between the water-saturated state and the non-saturated states would be even more pronounced than in Fig 4. Moreover, the true, absolute sorption hysteresis of the cell wall moisture would be larger than depicted in the same diagram.

On the other hand, the DSC method might overestimate the cell wall moisture content because water molecules close to solid surfaces can be restrained from freezing [49]. It could be argued that this layer of non-freezing water does not belong to the cell wall domain, and it is possible to estimate how much of the water in macro-voids that cannot freeze. For simplicity, assume cylindrical pores with radius, *R* (m) and a non-freezable surface layer of water with a thickness, *t* (m). In a cylindrical pore, the relative amount of non-freezable water could then be found as

$$\frac{m_{w,non-freezing}}{m_{w,total}}=\frac{\pi R^{2}-\pi {\left(R-t\right)}^{2}}{\pi R^{2}}=1-{\left(\frac{R-t}{R}\right)}^{2}$$ (5)

In the over-hygroscopic range, the macro-void pores in wood are filled or emptied based on their capillary radius as described previously. The investigated over-hygroscopic range covers 99.65–99.98% relative humidity and the fully water-saturated state which corresponds with capillary radii of 0.3–5.4 μm, again assuming cylindrical geometry. Thus, at 99.65% relative humidity, pores with a radius of 0.3 μm and below will be filled with water, while this is the case for pores of radius 5.4 μm at 99.98% relative humidity. A change from a relative humidity of 99.65% to 99.98% therefore only affects capillary water in pores of radius between 0.3 μm and 5.4 μm, since smaller macro-void pores are filled at both RH. In desorption, capillary water can be retained in much larger pores in this RH range if bottleneck pores of radius smaller than 0.3 μm are present. The relative amount of non-freezing surface water is larger in smaller pores. Therefore, as conservative estimate, we could assume that a change in observed freezable water between 99.65% and 99.98% relative humidity, either in absorption or desorption, is purely a result of a change in capillary water held in pores marginally larger than 0.3 μm. However, our data contains six conditioning levels in the over-hygroscopic range (including fully water-saturated). The potential overestimation of the change in cell wall moisture between these levels can therefore be calculated in each relative humidity step. This has been done in S3 Appendix by assuming a sizeable non-freezing surface water layer of 6 nm corresponding with 20 water molecules, i.e. thicker than the size of the water-induced pores within cell walls at saturation [44–46], and several times thicker than estimated sizes of non-freezable water layers in the literature which is typically in the range of 0.3–1 nm [50, 51]. The calculations show that the fraction of non-freezing water would be larger in smaller pores as evident in Eq 5. At 99.65% relative humidity, the threshold pore of radius 0.3 μm would contain 4% non-freezing surface water, while at 95% relative humidity the threshold pore of radius 21 nm would contain nearly 50% non-freezing water. The assignment of non-freezing water to cell wall water could therefore potentially overestimate the cell wall moisture content significantly. However, our data show that in wood conditioned at 95% relative humidity, no freezing water was detected at all, while wood conditioned at 99.65% RH contained capillary water corresponding with 0.1–0.3% moisture content. Even if all the detected freezing water at 99.65% RH were held in pores of radius 21 nm, i.e. the threshold pore of the next lower RH level of 95%, the overestimation in cell wall moisture would therefore only be 0.1–0.3% moisture content. In fact, between 99.98% RH and water-saturation the overestimation would be 0.2% moisture content due to the large difference in freezing water between these two levels, although the fraction of assumed non-freezing surface water is less than 0.3%.

To sum up, the DSC method employed to partition the total moisture content into cell wall water and capillary water could be inaccurate in this. However, if it contrary to literature results is believed that cell wall water may freeze, the true difference between the water-saturated and non-saturated states and the true cell wall sorption hysteresis would both be larger. On the other hand, if a thick non-freezing surface water layer is assumed, the error in the water partitioning would be insignificant. For these reasons, we are confident that the observed development in non-freezing and freezing water as function of relative humidity genuinely reflects that of cell wall water and capillary water, respectively.

## Conclusions

This study shows that sorption hysteresis in wood cell walls exist in the whole moisture range. The total absolute sorption hysteresis increased up to water-saturation, while the results indicated a decrease in sorption hysteresis in cell walls in the over-hygroscopic moisture range. Moreover, the results show that cell walls are not water-saturated before the wood is fully water-saturated which contradicts the long-held concept of the fibre saturation point as it was originally defined. Instead, the cell wall moisture content increases also when significant amounts of capillary water are present.

## Supporting information

S1 Appendix Additional data. Pressure plate data for thicker specimens and sorption isotherms (cell wall water and capillary water) for unextracted material.
(PDF)

S2 Appendix Information on data in S1 and S2 Datasets. (PDF)

S3 Appendix Error analysis. Calculations of errors related to a possible layer of non-freezing surface water.
(PDF)

S1 Dataset DSC data. Heat flow measured during melting of freezable water.
(ZIP)

S2 Dataset DSC and moisture content data. Cell wall water and capillary water in the over-hygroscopic range and at 95% RH, and moisture contents in the hygroscopic range.
(XLSX)
