# Supplementary material for: On sorption hysteresis in wood: Separating hysteresis in cell wall water and capillary water in the full moisture range
Source: PLoS One. 2019 Nov 15;14(11):e0225111. doi: 10.1371/journal.pone.0225111 (PMC6857914; doi:10.1371/journal.pone.0225111)
Supplement: S1 Appendix — Pressure plate data for thicker specimens and sorption isotherms (cell wall water and capillary water) for unextracted material. (PDF) [file pone.0225111.s001.pdf]

S1 Appendix. Additional Data. Pressure plate data for thicker specimens and sorption isotherm data (cell wall water and capillary water) for unextracted material

Maria Fredriksson[1]*, Emil Engelund Thybring[2]

[1] Division of Building Materials, Department of Building and Environmental Technology, Lund University, Lund, Sweden

[2] Biomass Science and Technology, Forest Nature and Biomass, Department of Geosciences and Natural Resource Management, University of Copenhagen, Frederiksberg, Denmark

Additional desorption isotherm measurements on thicker specimens

Additional pressure plate measurements were performed in order to:

1. verify that the thin specimen thickness influenced the over-hygroscopic desorption isotherm
2. ensure that the novel pressure plate system with smaller specimens and smaller cells gave the same results as conventional pressure plate equipment where larger specimens are used.

Douglas fir (*Pseudotsuga menziesii* (Mirb.) Franco) specimens with the dimensions 20 mm x 30 mm x 5 mm and 20 mm x 30 mm x 2 mm were cut from the same piece of wood as the specimens used in the paper. The smallest dimension (5 or 2 mm) was in the longitudinal direction. These specimens were extracted and vacuum saturated with water using the same procedure as described in the paper. Three specimens of each size were then conditioned at the following pressure levels: 0.92, 2.89, 4.78, 8.0 and 14.5 bar using standard pressure plate equipment (5 Bar and 15 Bar Pressure Plate Extractors, Soilmoisture Equipment Corp., Santa Barbara, CA, USA).

The results are shown in Figure S1.1 together with desorption data from Fig. 3. As expected, the desorption isotherm of the 5 mm specimens was higher than for the 2 mm specimens close to saturation. The reason for this is that 5 mm exceeds the length of the tracheids. The pits thus act as ink-bottle necks and cell lumen will be emptied when the applied pressure corresponds to the size of the pits openings. However, for the thinner specimens, the size of the lumen is decisive. Since the lumen is larger than the pit openings, cell lumina will be emptied at a lower pressure (i.e., at a higher relative humidity) for thin specimens.

Here, larger specimens were used compared to the ones used in the paper. The desorption isotherm obtained for these larger samples using standard pressure plate equipment was in line with the data obtained on smaller specimens using the novel pressure plate system. This confirms that accurate desorption data is obtained with the novel system with pressure plate cells of different design and considerably smaller specimens.

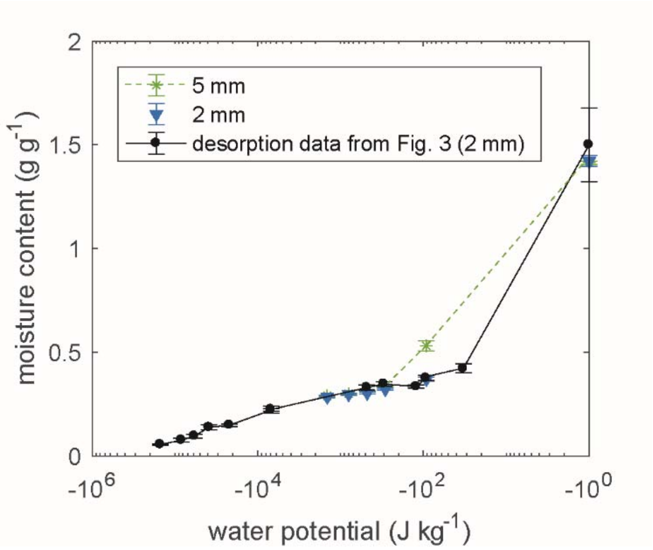

Figure S1.1 Pressure plate data. Additional pressure plate data for specimens of two thicknesses (5 mm and 2 mm) in the longitudinal direction together with data from Fig. 3 for smaller specimens with a thickness of 2 mm obtained using the novel pressure plate system. Data at water saturation is shown at -10^0 J kg^{-1}.

Data for unextracted specimens

All DSC measurements were additionally performed on unextracted material for the same moisture levels and by the same procedure of measurement as for the extracted material. Fig. S1.2 show total absorption and desorption isotherms. As for the extracted material, the results from the DSC measurements was used to separate the sorption isotherms into cell wall water and capillary water respectively (Fig. S1.3a). Corresponding absolute sorption hysteresis for cell wall water and capillary water respectively is shown in Fig. S1.3b. The total sorption hysteresis was almost non-existent in the upper most part of the sorption isotherm (Fig S1.2); the absorption and desorption isotherm merged. For the capillary water, the absorption isotherm was higher than the desorption isotherm at the highest moisture level, while for the cell wall water, the desorption isotherm was still higher than the absorption isotherm (Fig. S1.3b). The reason for the similar total desorption and absorption isotherms was thus the negative absolute sorption hysteresis in capillary water. Since the amount of capillary water was very small due to the thin specimens, the negative values in hysteresis here (Fig. S1.3b) should be interpreted with care.

As for the data for the extracted material presented in the paper, sorption hysteresis in cell wall water for the unextracted material remained at very high humidity levels. The difference in cell wall moisture content at water saturation and the other moisture levels in the over-hygroscopic moisture range is therefore not related to removal of extractives.

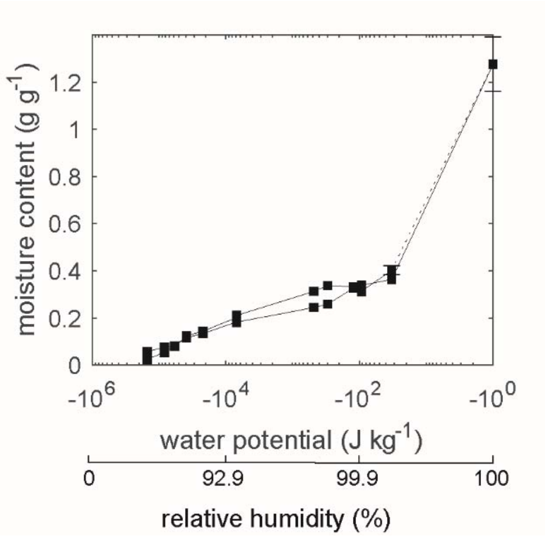

Figure S1.2 Total sorption isotherms for unextracted material. Total desorption and absorption isotherms for unextracted Douglas fir.

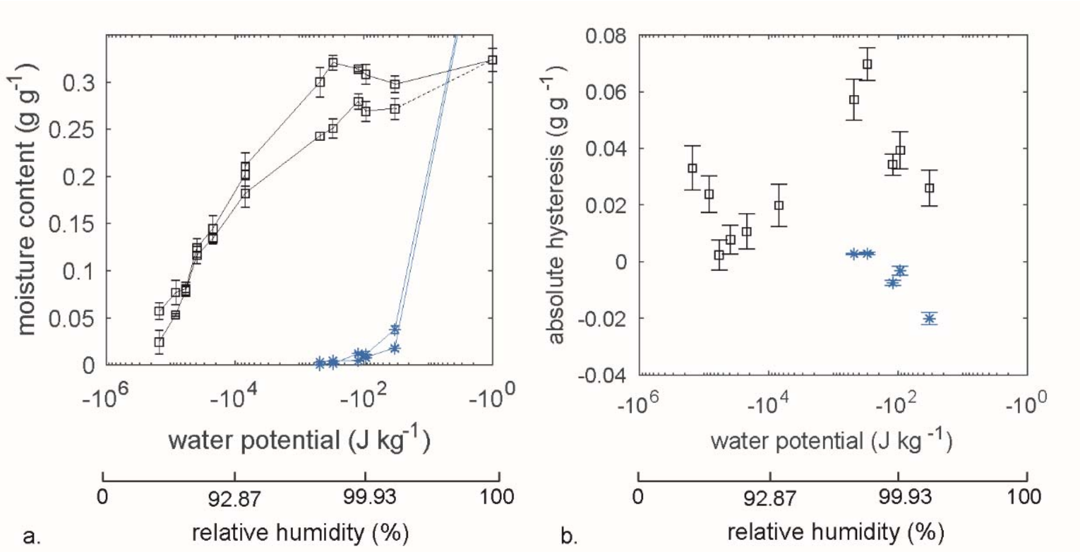

Figure S1.3 Separate sorption isotherms and sorption hysteresis. a. Separate sorption isotherms for cell wall water (black squares) and capillary water (blue asterisks) for unextracted Douglas fir. b. Absolute sorption hysteresis for cell wall water (black squares) and capillary water (blue asterisks) respectively. Note that at the two lowest moisture levels the amount of water in the sample was very small in relation to the resolution to the balance.
