# Supplementary material for: On sorption hysteresis in wood: Separating hysteresis in cell wall water and capillary water in the full moisture range
Source: PLoS One. 2019 Nov 15;14(11):e0225111. doi: 10.1371/journal.pone.0225111 (PMC6857914; doi:10.1371/journal.pone.0225111)
Supplement: S2 Appendix — (PDF) [file pone.0225111.s002.pdf]

S2 Appendix. Information about data in S1 Dataset and S2 Dataset.

Maria Fredriksson[1]*, Emil Engelund Thybring[2]

[1] Division of Building Materials, Department of Building and Environmental Technology, Faculty of Engineering, Lund University, Lund, Sweden

[2] Biomass Science and Technology, Forest Nature and Biomass, Department of Geosciences and Natural Resource Management, Faculty of Science, University of Copenhagen, Frederiksberg, Denmark

Attached data files

Experimental data obtained by Differential Scanning Calorimetry (DSC)

The DSC measurements were performed on a DSC Q2000 (TA Instruments, Eschborn, Germany) located at ETH Zürich, Switzerland. The instrument was programmed to perform the measurements by the following protocol:

1) Load sample
2) Sampling interval 0.10 s/pt
3) Data storage OFF
4) Equilibrate at -20.00 °C
5) Isothermal for 5.00 min
6) Data storage ON
7) Ramp 2.00 °C/min to 20.00 °C
8) Data storage OFF
9) Equilibrate at 40.00 °C
10) Remove sample
11) Repeat 1)-10)

The complete dataset for all DSC runs is found in "S1 Dataset" ("Absorption_DSC.tab", "Desorption_DSC.tab", and "Water-saturated_PureWater_DSC.tab").

Evaluated data

The evaluation of the integral heat of melting was found by integration of the heat flow in a temperature range visually picked for each DSC curve. The integrated heat flow as well as the masses and resultant moisture contents in total, in cell walls, and in macro-voids can be found in "S2 Dataset" in the sheet "Over-hygroscopic range". In the same xlsx-file, but in the sheet "Hygroscopic range", the moisture content of all specimens conditioned above saturated salt solutions can be found.
