# Supplementary material for: On sorption hysteresis in wood: Separating hysteresis in cell wall water and capillary water in the full moisture range
Source: PLoS One. 2019 Nov 15;14(11):e0225111. doi: 10.1371/journal.pone.0225111 (PMC6857914; doi:10.1371/journal.pone.0225111)
Supplement: S3 Appendix — Calculations of errors related to a possible layer of non-freezing surface water. (PDF) [file pone.0225111.s003.pdf]

S3 Appendix. Error analysis. Calculations of errors related to a possible layer of non-freezing surface water.

Maria Fredriksson[1]*, Emil Engelund Thybring[2]

[1] Division of Building Materials, Department of Building and Environmental Technology, Lund University, Lund, Sweden

[2] Biomass Science and Technology, Forest Nature and Biomass, Department of Geosciences and Natural Resource Management, University of Copenhagen, Frederiksberg, Denmark

Calculations

The influence of a non-freezing layer of water outside cell walls was investigated by assuming a non-freezing surface water layer of either 0.9 nm or 6 nm corresponding with 3 and 20 water molecules, respectively. For simplicity, cylindrical pores was assumed, and the relative amount of non-freezable water could then be found as

$$\frac{m_{\mathrm{w,non-freezing}}}{m_{\mathrm{w,total}}} = \frac{\pi R^2 - \pi (R-t)^2}{\pi R^2} = 1 - \left(\frac{R-t}{R}\right)^2 \qquad \text{(S3.1)}$$

where R (m) is pore radius and t (m) is the thickness of the non-freezable surface layer of water. For such cylindrical pores, the threshold pore radius in equilibrium (r_{eq}) with a given relative humidity (RH) φ (Pa Pa^{-1}), can be determined from:

$$\ln\varphi = -\frac{2\gamma\cos(\theta)V}{r_{\mathrm{eq}}RT} \qquad \text{(S3.2)}$$

Here, γ (N m^{-1}) is water surface tension, θ is water-wood surface contact angle, V (m^3 mol^{-1}) is molar volume of water, R (8.314 J mol^{-1} K^{-1}) is the gas constant, T (K) is temperature. The radii corresponding to the RH levels used in this study is given in Table S3.1. Similarly, the relative amount of non-freezing water given by Eq. S3.1 can be calculated for these pores based on the assumed thickness of the non-freezing water layer as seen in Table S3.1. It is clear that the relative amount is larger in smaller pores.

Table S3.1 Relative amount of non-freezing capillary water in capillaries filled at various levels of RH. Calculated from Eq. S3.1 using t = 6 nm and t = 0.9 nm corresponding to 20 and 3 water molecules, respectively.

Conditioning RH	r_{eq} (µm)	t (nm)	$m_{\mathrm{w,non-freezing}}/m_{\mathrm{w,total}}$ (%)	t (nm)	$m_{\mathrm{w,non-freezing}}/m_{\mathrm{w,total}}$ (%)
99.98 %	5.43	6	0.22	0.9	0.03
99.93 %	1.55	6	0.77	0.9	0.12
99.91 %	1.21	6	0.99	0.9	0.15
99.78 %	0.49	6	2.42	0.9	0.36
99.65 %	0.31	6	3.83	0.9	0.58
95.00 %	0.02	6	48.62	0.9	8.32

In the over-hygroscopic range, the macro-void pores in wood are filled or emptied based on their capillary radius as described in the main manuscript. In this study, the investigated over-hygroscopic range covers 99.65-99.98 % relative humidity and the fully water-saturated state which corresponds with capillary radii of 0.3-5.4 µm, again assuming cylindrical geometry. Thus, at 99.65 % relative humidity, pores with a radius of 0.3 µm and below will be filled with water, while this is the case for pores of radius 5.4 µm at 99.98 % relative humidity. A change from a relative humidity of 99.65 % to 99.98 % therefore only affects capillary water in pores of radius between 0.3 µm and 5.4 µm, since smaller macro-void pores are filled at both RH. In desorption, capillary water can be retained in much larger pores in this RH range if bottleneck pores of radius smaller than 0.3 µm are present.

The effect of non-freezing capillary water on the results obtained with DSC can be calculated from estimates of the size of voids being filled or emptied during a change in RH. For instance, the change in RH from 99.93 % to 99.91 % results in a decrease in the determined capillary water of 1.7 % moisture content, see the column ”difference in capillary moisture content” in Table S3.2. These levels of RH correspond with pore radii of 1.55 µm and 1.21 µm, respectively. As a conservative estimate, we assign the entire change in capillary water to emptying of voids marginally larger than the smallest pore size (1.21 µm) that have the highest relative amount of assumed non-freezing water (0.99 %), see Table S3.1. Given that the experimentally determined changed in capillary water is 1.7 % moisture content, we correct this quantity by normalisation with (100-0.99 %) to arrive at the true capillary water content under the assumption of a 6 nm thick non-freezable water layer in the water-filled voids. Similarly, we decrease the cell wall water with the amount added to the capillary water pool. As another example, in absorption the change from 95.00 % RH to 99.65 % RH is associated with a change in experimentally determined capillary water of 0.4 % moisture content. These levels of RH corresponds with pore radii of 0.02 µm and 0.31 µm, respectively. As a conservative estimate, we assign the entire change in capillary water to filling of voids marginally larger than the smallest pore size (0.02 µm) that have the highest relative amount of assumed non-freezing water (48.62 %). We then correct the determined capillary water by normalisation with (100-48.62 %), and decrease the cell wall water with the amount added to the capillary water pool. The results for all conditioning RH levels are summarized in Table S3.2 which shows that even for a sizeable assumed non-freezing water layer of 6 nm, the implications on the corrected cell wall water and capillary water are very minor. For the assumption of a 0.9 nm thick non-freezing water layer, the corrections are even more insignificant.

Table S3.2. Correction for assumed 6 nm layer thick non-freezing capillary water in the results for extracted Douglas fir samples. Numbers given in brackets are the standard deviations.

<table>
<tr><th></th><th>Conditioning RH</th><th>Cell wall moisture content (%)</th><th>Capillary moisture content (%)</th><th>Estimated relative amount of non-freezing water (%)</th><th>Difference in capillary moisture content (%)</th><th>Difference with non-freezing capillary water (%)</th><th>Corrected cell wall moisture content (%)</th><th>Corrected capillary moisture content (%)</th></tr>
<tr><td rowspan="14">Desorption</td><td rowspan="2">Water-saturated</td><td rowspan="2">33.4 (2.0)</td><td rowspan="2">79.2 (21.5)</td><td></td><td></td><td></td><td rowspan="2">33.3 (2.0)</td><td rowspan="2">79.4</td></tr>
<tr><td rowspan="2">0.22</td><td rowspan="2">75.8</td><td rowspan="2">76.0</td></tr>
<tr><td rowspan="2">99.98 %</td><td rowspan="2">30.7 (0.9)</td><td rowspan="2">3.4 (0.6)</td><td rowspan="2">30.7 (0.9)</td><td rowspan="2">3.4</td></tr>
<tr><td rowspan="2">0.77</td><td rowspan="2">0.9</td><td rowspan="2">0.9</td></tr>
<tr><td rowspan="2">99.93 %</td><td rowspan="2">31.3 (1.0)</td><td rowspan="2">2.5 (1.5)</td><td rowspan="2">31.3 (1.0)</td><td rowspan="2">2.5</td></tr>
<tr><td rowspan="2">0.99</td><td rowspan="2">1.7</td><td rowspan="2">1.7</td></tr>
<tr><td rowspan="2">99.91 %</td><td rowspan="2">30.3 (0.4)</td><td rowspan="2">0.8 (0.2)</td><td rowspan="2">30.3 (0.4)</td><td rowspan="2">0.8</td></tr>
<tr><td rowspan="2">2.42</td><td rowspan="2">-0.1</td><td rowspan="2">-0.1</td></tr>
<tr><td rowspan="2">99.78 %</td><td rowspan="2">31.4 (1.1)</td><td rowspan="2">0.8 (0.1)</td><td rowspan="2">31.4 (1.1)</td><td rowspan="2">0.9</td></tr>
<tr><td rowspan="2">3.83</td><td rowspan="2">0.5</td><td rowspan="2">0.5</td></tr>
<tr><td rowspan="2">99.65 %</td><td rowspan="2">31.6 (1.2)</td><td rowspan="2">0.3 (0.1)</td><td rowspan="2">31.2 (1.2)</td><td rowspan="2">0.7</td></tr>
<tr><td rowspan="2">48.62</td><td rowspan="2">0.3</td><td rowspan="2">0.7</td></tr>
<tr><td rowspan="2">95.00 %</td><td rowspan="2">22.4 (1.7)</td><td rowspan="2">0.0 (0.0)</td><td rowspan="2">22.4 (1.7)</td><td rowspan="2">0.0</td></tr>
<tr><td rowspan="2">48.62</td><td rowspan="2">0.4</td><td rowspan="2">0.7</td></tr>
<tr><td rowspan="12">Absorption</td><td rowspan="2">99.65 %</td><td rowspan="2">29.3 (1.8)</td><td rowspan="2">0.4 (0.1)</td><td rowspan="2">28.9 (1.8)</td><td rowspan="2">0.7</td></tr>
<tr><td rowspan="2">3.83</td><td rowspan="2">-0.1</td><td rowspan="2">-0.1</td></tr>
<tr><td rowspan="2">99.78 %</td><td rowspan="2">27.6 (2.3)</td><td rowspan="2">0.3 (0.1)</td><td rowspan="2">27.6 (2.3)</td><td rowspan="2">0.3</td></tr>
<tr><td rowspan="2">2.42</td><td rowspan="2">0.0</td><td rowspan="2">0.0</td></tr>
<tr><td rowspan="2">99.91 %</td><td rowspan="2">28.6 (0.5)</td><td rowspan="2">0.3 (0.0)</td><td rowspan="2">28.6 (0.5)</td><td rowspan="2">0.3</td></tr>
<tr><td rowspan="2">0.99</td><td rowspan="2">0.0</td><td rowspan="2">0.0</td></tr>
<tr><td rowspan="2">99.93 %</td><td rowspan="2">27.5 (1.3)</td><td rowspan="2">0.3 (0.2)</td><td rowspan="2">27.5 (1.3)</td><td rowspan="2">0.3</td></tr>
<tr><td rowspan="2">0.77</td><td rowspan="2">-0.2</td><td rowspan="2">-0.2</td></tr>
<tr><td rowspan="2">99.98 %</td><td rowspan="2">27.6 (1.1)</td><td rowspan="2">0.1 (0.0)</td><td rowspan="2">27.6 (1.1)</td><td rowspan="2">0.1</td></tr>
<tr><td rowspan="2">0.22</td><td rowspan="2">79.1</td><td rowspan="2">79.3</td></tr>
<tr><td rowspan="2">Water-saturated</td><td rowspan="2">33.4 (2.0)</td><td rowspan="2">79.2 (21.5)</td><td rowspan="2">33.3 (2.0)</td><td rowspan="2">79.4</td></tr>
<tr><td></td><td></td><td></td></tr>
</table>
